# Clinical Features of Nontuberculous Mycobacterial Pulmonary Disease in the Yangtze River Delta of China: A Single-Center, Retrospective, Observational Study

**DOI:** 10.3390/tropicalmed8010050

**Published:** 2023-01-09

**Authors:** Hai Lou, Ansheng Zou, Xiaona Shen, Yong Fang, Qin Sun, Fen Zhang, Wei Sha

**Affiliations:** 1 Shanghai Key Laboratory of Tuberculosis, Tuberculosis Diagnosis and Treatment Center, Shanghai Pulmonary Hospital, Tongji University, Shanghai 200433, China; 2 Intensive Care Unit, Yantai Qishan Hospital, Yantai 264001, China; 3 Department of Pulmonary and Critical Care Medicine, Shanghai Forth People’s Hospital, School of Medicine, Tongji University, Shanghai 200434, China

**Keywords:** clinical features, *M. abscessus*, *M. intracellulare*, *M. kansasii*, nontuberculous mycobacteria, pulmonary disease

## Abstract

With increased focus on nontuberculous mycobacterial pulmonary disease (NTM-PD), and the improvement in detection methods, the global incidence continues to increase every year, but the diagnosis and treatment are difficult with a high misdiagnosis rate and poor curative effect. This study aimed to analyze the clinical indicators of different pathogenic NTM in the Yangtze River Delta. The study retrospectively analyzed the medical records of patients with NTM-PD, who resided in the Yangtze River Delta and were diagnosed using sputum or bronchial lavage fluid and hospitalized in Shanghai Pulmonary Hospital from March 2017 to February 2019. The clinical data of confirmed patients were collected. Among the 513 cases of NTM-PD, 482 cases were infected by four common bacteria: *Mycobacterium intracellulare* (224, 46.5%), *M. abscessus* (138, 28.6%), *M. kansasii* (84, 17.4%), and *M. avium* (36, 7.5%). The analysis found that different NTM strains have their corresponding positive and negative correlation factors (*p* < 0.05). *M. intracellulare*, *M. abscessus*, *M. kansasii*, and *M. avium* were the main pathogenic bacteria isolated from patients with NTM-PD in the Yangtze River Delta were. Different strains resulted in different clinical features, assisting in the early diagnosis and treatment of NTM-PD.

## 1. Introduction

Nontuberculous mycobacteria (NTM) refers to the general name of mycobacteria other than Mycobacterium tuberculosis complex, *M. leprae complex*, and *M. ulcerans* [1]. They can invade the lung, lymph node, bone, joint, skin, soft tissue, and other tissues and organs, and cause systemic disseminated diseases; the lung is the most frequently involved part [2,3]. Some studies showed that NTM were conditional pathogens, and the pathogenicity to humans was lower than that of *M. tuberculosis*. However, NTM could cause diseases in the case of susceptible factors that impaired the local or systemic immune function of the host. Environmental pollutants and infected secretions could also cause the spread of the disease [4,5,6]. Since NTM and *M. tuberculosis* could not be identified only by the sputum smear examination, and the clinical symptoms and imaging features of NTM pulmonary disease were similar to those of pulmonary tuberculosis, it was quite common for NTM to be misdiagnosed as tuberculosis [7,8,9]. The incidence of NTM has increased at an alarming rate with increased understanding on NTM in recent years [3,10,11], and the harm has gradually been noticed [3]. However, NTM strains are many, and the time of bacterial culture is long. It is particularly difficult to distinguish the NTM strains that cause diseases in infected patients and their distribution, and relying only on the culture results before treatment intervention may delay the treatment of the disease. If warning for the diagnosis can be obtained in the early stage of the disease course through clinical manifestations, laboratory examination results, and imaging characteristics of the patients, it may be helpful for the patient’s condition.

At present, clinical studies on NTM pulmonary disease are few, and the number of cases is small. Most of them focus on epidemiology and drug sensitivity. This study included in-patients with NTM pulmonary disease diagnosed using sputum or lavage fluid from the Yangtze River Delta and hospitalized in Shanghai Pulmonary Hospital. It analyzed the distribution of NTM strains and compared the clinical manifestations, laboratory examination results, imaging characteristics, and drug sensitivity test results of patients infected by different NTM strains so as to provide a deeper understanding of the clinical characteristics of NTM pulmonary disease.

## 2. Materials and Methods

### 2.1. Research Participants

The clinical data of 3803 patients with positive mycobacterial culture in sputum or bronchial lavage fluid hospitalized in Shanghai Pulmonary Hospital from March 2017 to February 2019 were collected retrospectively (the long-term residence of hospitalized patients was mainly in the Yangtze River Delta, including Jiangsu province, Zhejiang province, Shanghai, and Anhui province). The inclusion criteria were according to the 2020 ATS/ERS/ESCMID/IDSA Clinical Practice Guidelines: treatment of NTM pulmonary disease [1]. The mycobacteria in positive culture specimens of sputum or bronchoalveolar lavage fluid were identified by reverse dot-blotting hybridization with polymerase chain reaction (PCR), and 513 patients with NTM pulmonary disease were confirmed. The exclusion criteria were as follows: (1) Breastfeeding or pregnant women and patients complicated with severe organic diseases, malignant tumors, or HIV-positive patients; (2) patients with mixed infection; (3) patients with rare bacteria; and (4) patients with incomplete clinical data. Finally, 482 cases were enrolled. This study was approved by the ethics committee of Shanghai Pulmonary Hospital, and the informed consent was exempted by the ethics committee (K20-016).

### 2.2. Serum T-SPOT Test

In this study, the γ-interferon release test kit (Oxford Immtmotee, Abingdon, UK) was used. We added 50 μL culture-medium (negative control), positive control, tuberculosis specific antigen A and B to the corresponding test holes. Each hole was added with 100 μL cell suspension, incubated in incubation bath of 36 °C, 50% CO_2_, 90% humidity incubator for 20~24 h. Additionally, the plate should be washed with PBS for 4 times. Then, 50 μL working enzyme complex solution was added into each hole, incubating at 5 °C for 60 min, washing the plate with 200 μL PBS for 4 times, and adding 50 μL BCIP/NBT substrate solution into each test hole. It was left at room temperature for 7 min and each hole was washed with distilled water, and observed after drying. If the number of negative control spots was 0~5, and the number of A or B holes was ≥6, or the number of negative holes was ≥6, and the number of A or B holes was ≥2 times the number of negative holes, it was judged to be positive. If the number of A and B holes was not enough, and the positive control response was good, then it was judged to be negative.

### 2.3. Serum TB Antibody Test

In this study, the Mycobacterium tuberculosis antibody diagnostic kit (Colloidal gold method) (Shanghai Aopu Biological Medicine Co., Ltd., Shanghai, China) was used. The specific membrane protein antigen of Mycobacterium tuberculosis was isolated and purified by dot immunocolloidal gold filtration (DIGFA), then sampled and solidified on the nitrate cellulose membrane. The TB antigen on the membrane captured the mycobacterium tuberculosis antibodies in human serum samples. The captured TB IgG antibodies can be colored by Staphylococcal A protein (SPA) colloidal gold conjugate (SPA can specifically bind to IgG) to form red spots. Positive and negative results can be determined according to the presence of red spots, so as to determine the presence of Mycobacterium tuberculosis antibodies.

### 2.4. NTM Strain Identification and Drug Sensitivity Test

According to the China Specification for Bacteriological Examination of Tuberculosis, the respiratory samples (sputum or BALF) of the patients were cultured and identified by the BACTEC MGIT 960 method. They were cultured and identified as NTM at least twice. After that, the drug sensitivity tests of streptomycin, isoniazid, rifampicin, ethambutol, amikacin, capreomycin, and ofloxacin were carried out by the proportional method strictly following the BD Company (USA) “MGIT Operation Procedure Guidelines”, and the specialized personnel of the laboratory department were responsible for the operation.

Further molecular detection was performed on the samples of strains identified as NTM, and PCR reverse dot-blotting hybridization was used for identifying mycobacterial strains following the instructions of the Mycobacterium Identification Gene Detection Kit produced by Yaneng BioSciences Co., Ltd. (Shenzhen, China), batch No.: M20140401.

### 2.5. Study Design

This retrospective case–control study was conducted to collect the general demographic characteristics, clinical symptoms, laboratory examination indicators, imaging manifestations, and drug sensitivity results of the enrolled cases. The imaging manifestations included morphology (strip shadow, patchy shadow, tree-in-bud sign, nodule shadow, pleural thickening or pleural effusion, mediastinal lymph node enlargement or calcification, emphysema or bullae, bronchiectasis, cavity, centrilobular tubercle) and the range of involved lung fields. The aforementioned data were respectively statistically analyzed. The imaging manifestations were interpreted by two imaging physicians with Deputy High Professional Title and by a third imaging physician with Senior Professional Title in the case of disagreement.

### 2.6. Statistical Analysis

SPSS 22.0 software (IBM, Armonk, NY, USA) was used for statistical analysis in this study. The continuous variables were expressed as mean ± standard deviation, which conformed to a normal distribution using the Shapiro–Wilk (S-W) method. Age, BMI, hemoglobin level, leukocyte count, albumin level, and erythrocyte sedimentation rate (ESR) were compared by one-way analysis of variance. Chest CT, sex, T-Spot, MTB antibody, clinical symptoms, and previous history were expressed as the rate or constituent ratio and compared using the multi-group chi-square test. When the expected count value was less than five cases, Fisher’s exact test was used for correction, and a *p* value < 0.05 indicated a statistically significant difference. R software (version 4.1.1; R Development Core Team, Vienna, Austria) was used for correlation and ROC analyses, and the statistically significant indicators of each group were used as joint variables for multivariate ROC analysis. The correlation coefficient was calculated by the Pearson/Spearman method, and the heat map was drawn using the R language pheatmap function.

## 3. Results

### 3.1. Distribution Proportion of NTM Pulmonary Disease Strains in the Yangtze River Delta

**Figure 1:**
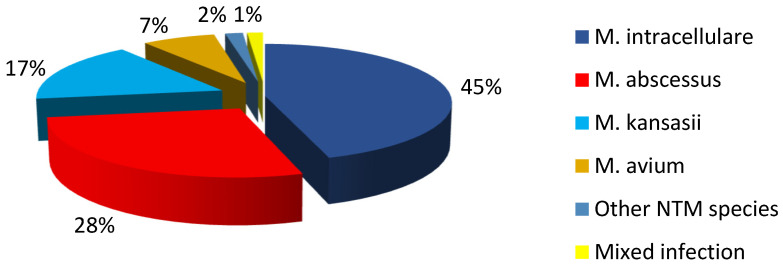
Distribution proportion of NTM pulmonary disease strains in the Yangtze River Delta.

**Figure 2:**
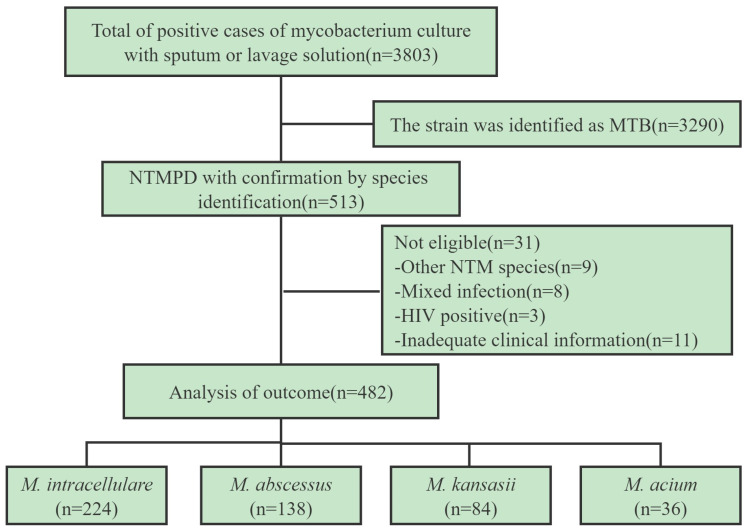
Screening flow chart.

From March 2017 to February 2019, 513 patients (13.5%) were diagnosed with nontuberculous mycobacterial pulmonary disease and hospitalized in Shanghai Pulmonary Hospital. They were infected by *M. intracellulare* (231, 45.0%), *M. abscessus* (142, 28%), *M. kansasii* (86, 17%), *M. avium* (37, 7%), and other rare strains (9, 2%) and had mixed infection (8, 1%) (Figure 1). Further, there were 9 cases of rare NTM strains: *M. scrofulaceum* (3), *M. fortuitum* (2), *M. chelonae* (2), *M. xenopi* (1) and *M. szulgai* (1); 8 cases of mixed infection: *M. tuberculosis* with NTM (3), *M. abscessus* with *M. intracellulare* (2), *M. abscessus* with *M. avium* (2), and *M. scrofulaceum* with *M. intracellulare* (1); 3 cases with HIV positivity: *M. intracellulare* (2) and *M. abscessus* (1); and 11 cases with incomplete clinical data: *M. intracellulare* (5), *M. abscessus* (3), *M. kansasii* (2), and *M. avium* (1). A total of 482 patients with NTM pulmonary disease were finally included in this study, including *M. intracellulare* (224, 46.5%), *M. abscessus* (138, 28.6%), *M. kansasii* (84, 17.4%), and *M. avium* (36, 7.5%) (Figure 1 and Figure 2).

### 3.2. Clinical Characteristics and Laboratory Indicators of Different NTM Cases

Among 482 patients with NTM pulmonary disease, 224 were in the *M*. *intracellulare* group, 138 cases in the *M*. *abscessus* group, 84 cases in the *M*. *kansasii* group, and 36 cases in the *M*. *avium* group. The general demographic characteristics, clinical symptoms, and laboratory examination indicators of patients in the four groups were statistically analyzed. Statistically significant differences were found in age, sex distribution, previous history of pulmonary tuberculosis and bronchiectasis, BMI, symptoms [cough duration (≥6 months), hemoptysis, emaciation], and indicators (T-Spot, tuberculosis antibody, ESR) (all *p* < 0.05). However, no significant statistically significant difference was found in the history of smoking, previous history of chronic obstructive pulmonary disease (COPD), bronchial asthma, or chronic bronchitis, symptoms (cough, expectoration, fever, fatigue, chest tightness, and chest pain), or indicators (hemoglobin, leukocyte, and albumin) (all *p* > 0.05) (Table 1).

### 3.3. Chest Imaging Manifestations of Different NTM Cases

The imaging manifestations of 482 patients with NTM-PD are shown in Table 2. Statistically significant differences were found in imaging features such as patchy shadow, pleural thickening or pleural effusion, mediastinal lymph node enlargement or calcification, bronchiectasis, and cavity and number of involved lung fields (>3) (all *p* < 0.05). However, no significant difference was found in imaging manifestations such as strip shadow, tree-in-bud sign, nodule shadow, emphysema, pulmonary bullae, or centrilobular tubercle (all *p* > 0.05).

### 3.4. BACTEC MGIT 960 Culture and Drug Sensitivity Test Results of Four Groups of Mycobacteria

**Figure 3:**
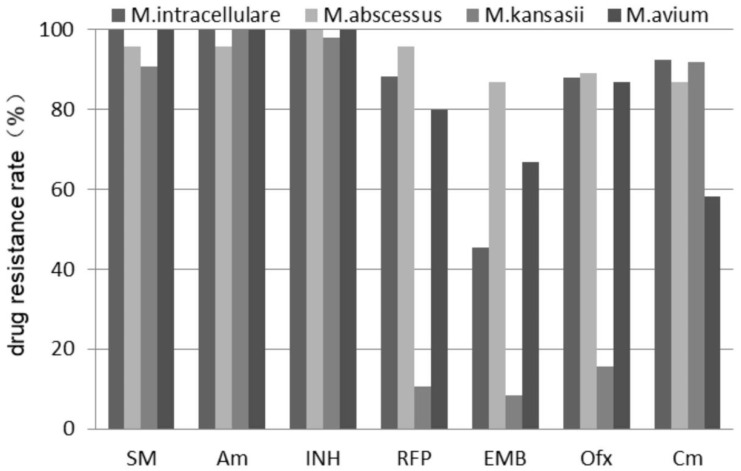
Drug resistance rates of patients in different groups to common anti-tuberculosis drugs. Note: Am: amikacin; Cm: capreomycin; EMB: ethambutol; INH: isoniazid; Ofx: ofloxacin; RFP: rifampicin; SM: streptomycin.

The drug sensitivity test results of NTM-PD strains in 482 cases showed that the drug resistance rates to seven common anti-tuberculosis drugs were all high (Figure 3). The resistance rates in the *M*. *kansasii* group to rifampicin, ethambutol, and ofloxacin were relatively low, which were 10.7% (9/84), 8.3% (7/84), and 15.5% (13/84), respectively. The resistance rate in the *M*. *intracellulare* group to ethambutol was 45.5% (102/224). The resistance rate in the *M*. *avium* group to ethambutol and capreomycin was 66.7% (24/36) and 58.3% (21/36), respectively. The drug resistance rates in the *M*. *abscessus* group to the aforementioned anti-tuberculosis drugs were all higher than 85%.

### 3.5. Correlation Analysis of the Clinical Indicators of Cases with Different NTM Pulmonary Diseases

**Figure 4:**
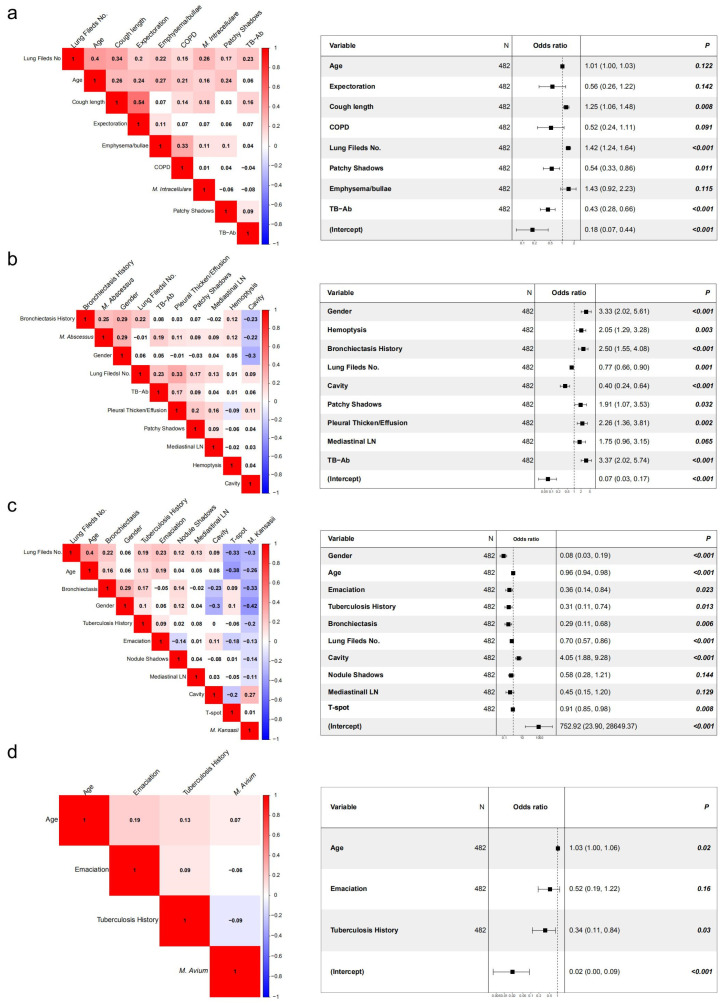
(**a**) The results of correlation analysis in the *M. intracellulare* group: the *M*. *intracellulare* pulmonary disease was positively correlated with the duration of cough and the number of involved lung fields on imaging, and negatively correlated with positive blood tuberculosis antibody and patchy shadow on imaging (all *p* < 0.05). (**b**) The results of correlation analysis in the *M*. *abscessus* group: *M*. *abscessus* pulmonary disease was positively correlated with female sex, hemoptysis, previous history of bronchiectasis, positive blood tuberculosis antibody, patchy shadow on imaging, and pleural thickening or pleural effusion, and negatively correlated with cavity on imaging and the number of involved lung fields (all *p* < 0.05). (**c**) The results of correlation analysis in the *M*. *kansasii* group: *M*. *kansasii* pulmonary disease was positively correlated with positive T-Spot and cavity on imaging, and negatively correlated with female, age, emaciation, previous history of pulmonary tuberculosis, bronchiectasis, and the number of involved lung fields on imaging (all *p* < 0.05). (**d**) The results of correlation analysis in the *M*. *avium* group: *M*. *avium* pulmonary disease was positively correlated with age and negatively correlated with the previous history of pulmonary tuberculosis (all *p* < 0.05).

The correlation analysis was conducted on the clinical indicators of 482 patients with NTM pulmonary disease caused by four common strains. The results were as follows (Figure 4).

### 3.6. AUC Analysis on Correlation Matrix Models of Mycobacteria in Four Groups

The variables related to the diagnosis of NTM were obtained from the aforementioned correlation analysis, and *p* < 0.05 indicated a statistically significant difference. However, the absolute *R* values were all low, indicating that although the relevant variables obtained had a certain distinguishing effect, they were not enough to be used as a single specific indicator. Hence, a comprehensive analysis should be combined with other clinical features. Therefore, the variables related to NTM diagnosis obtained from the aforementioned correlation analysis were further used in a multi-class variable analysis so as to predict the possible diagnostic criteria of NTM through multi-class variable analysis.

**Figure 5:**
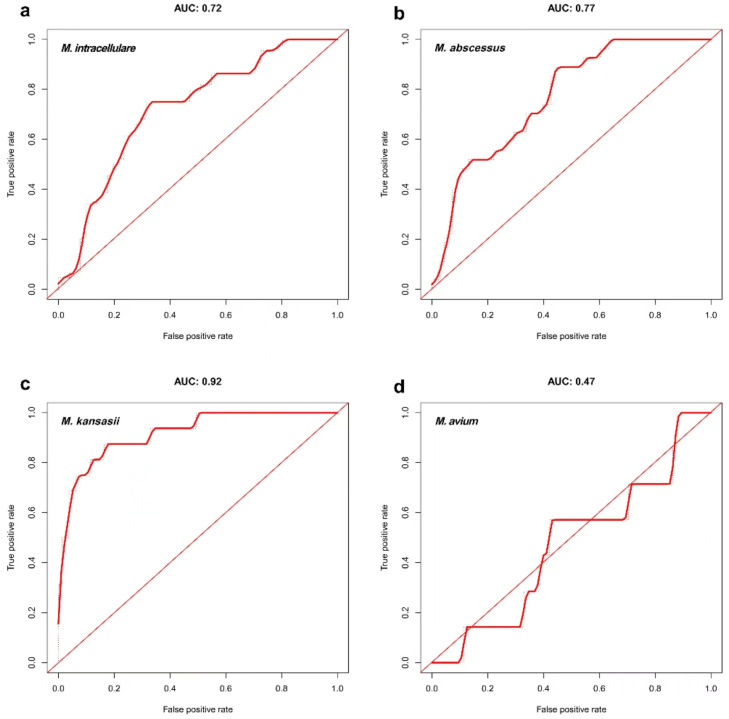
AUC curve of correlation models of mycobacteria in the four groups. (**a**). Duration of cough, positive blood tuberculosis antibody, patchy shadow on imaging, and the number of involved lung fields were taken as joint variables. The strain was identified as *M*. *intracellulare*, and the obtained AUC value was 0.72. (**b**). Sex, hemoptysis, previous history of bronchiectasis, positive blood tuberculosis antibody, patchy shadow on imaging, pleural thickening or pleural effusion, cavity, and the number of involved lung fields were taken as joint variables. The strain was identified as *M*. *abscessus*, and the obtained AUC value was 0.77. (**c**). Sex, age, emaciation, previous history of pulmonary tuberculosis, positive T-Spot, cavity on imaging, bronchiectasis, and the number of involved lung fields were taken as joint variables. The strain was identified as *M*. *kansasii*, and the obtained AUC value was 0.92. (**d**). Age and previous history of pulmonary tuberculosis were taken as joint variables. The strain was identified as *M*. *avium*, and the obtained AUC value was 0.47.

The AUC analysis of the four groups of models showed that the AUC values in the *M*. *intracellulare*, *M*. *abscessus*, and *M*. *kansasii* groups were all higher than 0.7. The value in the *M*. *kansasii* group was the highest, reaching 0.92, and that in the *M*. *avium* group was the lowest (0.47). This might be related to the small number of cases in *M*. *avium* group (Figure 5a–d).

## 4. Discussion

The incidence rate of NTM pulmonary disease continues to increase every year with the progress of diagnostic technology (such as gene sequencing) and the improvement in public understanding of the harmfulness of NTM pulmonary disease. The incidence and prevalence of NTM pulmonary disease are higher than those of tuberculosis in some countries with a low burden of tuberculosis [3,12,13,14]. In the United States, the annual incidence of NTM-PD increased from 20 cases per 100,000 people in 1997 to 47 cases per 100,000 people in 2007. During the same period, the prevalence in each state was 112 cases per 100,000 people, and the highest was 396 cases per 100,000 people in Hawaii [15]. A large number of patients had tuberculosis in China, and the incidence of NTM-PD is also significantly higher than that in other countries. Therefore, it is of great importance to understand more about the clinical characteristics of related pathogens and possible effective therapeutic drugs. This study found that different NTM strains had their own characteristics in terms of patients’ age, sex, symptoms, test results, and imaging manifestations, which could help better understand NTM pulmonary disease. This study also further analyzed the drug sensitivity results and found that the *M*. *kansasii* group had relatively low resistance to rifampicin, ethambutol, and quinolones, while the other three groups of mycobacteria had higher resistance to various anti-tuberculosis drugs, which could guide the diagnosis and treatment of NTM-PD. Actually, the BACTEC MGIT 960 ratio method has defects in drug sensitivity of mycobacteria such as *M. intracellulare, M. abscessus and M. avium*. Additionally, the sensitivity of macrolides cannot be evaluated by the BACTEC MGIT 960 ratio method. However, this study was a retrospective study, during March 2017 to February 2019, and Tuberculosis culture and drug sensitivity were performed by the BACTEC MGIT 960 ratio method in Shanghai Pulmonary Hospital.

The distribution of NTM strains was different in different countries due to the differences in living habits, temperature, and humidity. This study found that the isolation rate of NTM was about 13.5% among the patients with positive sputum or lavage fluid culture hospitalized in Shanghai Pulmonary Hospital (mainly resided in the Yangtze River Delta such as Jiangsu province, Zhejiang province, Shanghai, and Anhui province). The pathogenic bacteria isolated from NTM pulmonary disease were *M*. *intracellulare* (45%), *M*. *abscessus* (28%), *M*. *kansasii* (17%), *M*. *avium* (7%), other rare strains (2%), and mixed types (1%), which were consistent with the most commonly isolated pathogenic NTM reported in Asia, which *M. avium–intracellulare* complex (MAC), *M*. *abscessus*, and *M*. *kansasii* successively [16,17,18]. In Europe and North America, MAC, *M*. *gordonae*, *M*. *xenopi*, and *M*. *fortuitum* were the most common, while in South America, MAC, *M*. *kansasii*, *M*. *gordonae*, and *M*. *fortuitum* were the most common [19].

In this study, various clinical indicators of different NTM strains were analyzed. It was found that patients with *M*. *intracellulare* had a long duration of cough (lasting for more than 6 months, accounting for 71%), low positive rate of blood tuberculosis antibody, wide range of involved lung fields (more than three lung fields, accounting for 83%), and few patchy shadows. Due to the relatively small number of enrolled cases, the patients with *M*. *avium* pulmonary disease had fewer statistically significant correlation indicators, which were positively correlated with age and negatively correlated with the previous history of pulmonary tuberculosis. However, the patients in the *M*. *intracellulare* and *M*. *avium* groups were of high age and had a long disease course and extensive lesion involvement, which are consistent with those reported by Sadamatsu et al. [20]. These two were collectively referred to as MAC. Andréjak et al. [21] suggested that patients with MAC usually had some basic diseases such as COPD, bronchiectasis, and tuberculosis due to their slightly high age. As the autoimmune function of these patients was low and not enough to resist MAC infection, the clinical incidence was high, which is consistent with the fact that it had the largest number of corresponding cases in this study. The patients in the *M*. *abscessus* group were mainly female (78.3%), and the symptom of hemoptysis was common. Most of them had a history of bronchiectasis and a high positive rate of blood tuberculosis antibody (65.9%). The imaging manifestations of tree-in-bud signs and patchy shadows were common, which were consistent with those reported by Chu et al. [22]. This study also suggested that pleural thickening or pleural effusion was common, cavities were rare, and the range of involved lung fields was relatively limited. Patients in the *M*. *kansasii* group were mainly male (90.5%) with low age, history of pulmonary tuberculosis or bronchiectasis, low incidence of emaciation, and short duration of cough, which were similar to the results reported by Park et al. [23]. These might be related to their low age, short duration of cough, and acceptable general conditions. The positive rate of T-Spot in the *M*. *kansasii* pulmonary disease group was significantly higher than that in other groups, which was consistent with the results reported by Johnston et al. [24]. This might be related to the overlapping between the stimulating antigens (Early Secreted Antigenic Target 6, ESAT-6 and Culture Filter Protein 10, CFP-10 antigens) used in the T-Spot detection method and the antigens of four NTM (*M*. *kansasii*, *M*. *marinum*, *M*. *szulgai*, and *M*. *gordonae*). Therefore, when patients were infected by these NTM, T-Spot test results might also be positive. The imaging manifestations of *M*. *kansasii* pulmonary disease were difficult to be distinguished from those of *M*. *tuberculosis*, and both lesions were mainly cavitary [25]. This study also showed similar results. In the imaging manifestations in the *M*. *kansasii* group, the lung lesions were relatively limited, and the main manifestation was cavity (85.7% in this study, significantly higher than those in other groups). At the same time, bronchiectasis was rare.

This study found that the positive rate of serum TB antibody was higher in the *M. abscess* group, but lower in the *M. intracellular* group. The sensitivity and specificity of serum TB antibody varied widely, which could be 47%–79% and 70%–93% in active tuberculosis respectively [26,27]. Further research is needed to obtain more accurate results.

In this study, the resistance of four common NTM strains to seven anti-tuberculosis drugs was also analyzed. The results showed that the four common NTM strains had high resistance rates to first-line anti-tuberculosis drugs, which is consistent with the reports of Kwon and Zhou et al. [28,29]. The mechanism of drug resistance is complex, including natural cell wall barrier, drug pumping system, inactivation of drugs, variation or deletion of drug targeting sites, and so forth, and may also be the result of the combined action of the aforementioned factors [30]. Among the four NTM strains, *M. kansasii* had relatively low resistance to rifampicin, ethambutol, and quinolones, which also explained the good curative effect and a short course of treatment of *M*. *kansasii* pulmonary disease after patients received the standard anti-tuberculosis regimen [31]. The drug resistance rates of the other three mycobacteria to the aforementioned seven anti-tuberculosis drugs were high, especially *M*. *abscessus*, whose drug resistance rates were all higher than 85%, and the corresponding clinical treatment effect was also poor, which is consistent with the relevant literature [32].

The aforementioned clinical characteristics and drug sensitivity results may provide certain help to clinical work, but still some limitations existed. This study was a single-center retrospective observational study. The selected cases were all in-patients, and the sample sizes of some groups were small. Besides the four NTM pathogenic bacteria mentioned earlier, other strains were rare, which might be related to the regional differences: most patients in this study were from Jiangsu province, Zhejiang province, Shanghai, and Anhui province. Based on the aforementioned factors, the results of this study might be biased. It is hoped that these results could be further verified by large, multi-center, prospective cohort studies in the future.

In conclusion, the main pathogenic bacteria isolated from patients with NTM pulmonary disease in the Yangtze River Delta were *M*. *intracellulare*, *M*. *kansasii*, *M*. *abscessus*, and *M*. *avium*, and the clinical characteristics of each strain were different. If T-Spot was positive for young and middle-aged male patients with a short course of the disease, low age, and normal body shape, the imaging lesions were relatively limited, mainly cavitary, and less complicated with bronchiectasis. The preliminary identification of strains suggested NTM, and the possibility of *M*. *kansasii* pulmonary disease should be first considered. Also, the anti-tuberculosis treatment effect was good. Patients with *M*. *abscessus* pulmonary disease were mainly female, and commonly had hemoptysis and positive blood tuberculosis antibody. The lesions were mostly patchy shadows complicated with pleural thickening or pleural effusion, the cavities were rare, and the scope of the lesions involved was relatively limited. Patients with *M*. *intracellulare* pulmonary disease and *M*. *avium* pulmonary disease often were old aged and had a long duration of cough, negative blood tuberculosis antibody, and extensive lesion involvement. Except for *M*. *kansasii*, the other three NTM strains had high drug resistance rates to anti-tuberculosis drugs and poor effects of anti-tuberculosis treatment.

Our study comprehensively described the clinical characteristics of the common pathogenic strains of NTM from the aspects of general demographic characteristics, clinical symptoms, test results, strain identification, imaging characteristics, drug sensitivity results, and so forth, which provides some help for the early diagnosis and treatment of NTM pulmonary disease.

## Figures and Tables

**Table 1:** Baseline data of patients with NTM pulmonary disease in four groups.

Baseline Data	*M*. *intracellulare* Group (224 Cases)	*M*. *abscessus* Group (138 Cases)	*M*. *kansasii* Group (84 Cases)	*M*. *avium* Group (36 cases)	Test Value	*p* Value
Age (years, mean ± SD)	60 ± 14	58 ± 14	48 ± 16	57 ± 15	12.49	<0.001
Sex (cases, %)						
Male	93 (41.5)	30 (21.7)	76 (90.5)	16 (44.4)	101.60	<0.001
Female	131 (58.5)	108 (78.3)	8 (9.5)	20 (55.6)		
Previous history (cases, %)						
Pulmonary tuberculosis	78 (34.8)	48 (34.8)	8 (9.5)	6 (16.7)	24.04	<0.001
Chronic obstructive pulmonary disease (COPD)	18 (8.0)	11 (8.0)	7 (8.3)	2 (5.6)	0.30	0.96 *
Bronchiectasia	105 (46.9)	90 (65.2)	8 (9.5)	15 (41.7)	65.92	<0.001
Bronchial asthma	27 (12.1)	15 (10.9)	7 (8.3)	2 (5.6)	1.58	0.666 *
Chronic bronchitis	36 (16.1)	12 (8.7)	8 (9.5)	7 (19.4)	6.34	0.092 *
Smoking history (cases, %)	141 (62.9)	78 (56.5)	48 (57.1)	17 (47.2)	3.97	0.266
BMI (kg/m^2^)	18.9 ± 3.0	19.4 ± 3.1	20.1 ± 2.8	19.6 ± 3.1	3.05	0.029
Symptoms (cases, %)						
Cough	210 (93.8)	131 (94.9)	74 (88.1)	35 (97.2)	4.43	0.202 *
Duration of cough (≥6 months)	159 (71.0)	92 (66.7)	23 (27.4)	20 (55.6)	51.60	<0.001
Expectoration	203 (90.6)	129 (93.5)	72 (85.7)	34 (94.4)	4.00	0.254 *
Fever	50 (22.3)	34 (24.6)	18 (21.4)	7 (19.4)	0.61	0.895
Hemoptysis	80 (35.7)	70 (50.7)	23 (27.4)	12 (33.3)	14.25	0.002
Fatigue	148 (66.1)	95 (68.8)	47 (56.0)	22 (61.1)	4.24	0.237
Chest tightness	146 (65.2)	92 (66.7)	44 (52.4)	22 (61.1)	5.37	0.147
Chest pain	48 (21.4)	32 (23.2)	15 (17.9)	6 (16.7)	1.33	0.721
Emaciation	68 (30.4)	38 (27.5)	12 (14.3)	6 (16.7)	10.05	0.018
Laboratory examination (mean ± SD)						
Positive T-Spot (cases, %)	52 (23.2)	42 (30.4)	48 (57.1)	11 (30.6)	32.67	<0.001
Positive serum TB antibody (cases, %)	104 (46.4)	91 (65.9)	34 (40.5)	16 (44.4)	18.54	<0.001
Hemoglobin (g/L)	120.2 ± 18.1	119.8 ± 15.7	121.4 ± 15.6	119.3 ± 24.5	0.19	0.906
Leucocyte (×10^9^/L)	6.2 ± 2.2	6.3 ± 2.9	6.6 ± 2.3	5.9 ± 1.9	0.86	0.461
Albumin (g/L)	37.2 ± 4.7	37.9 ± 4.4	37.2 ± 6.6	37.9 ± 4.4	0.87	0.461
ESR (mm/H)	44.4 ± 33.7	38.6 ± 29.7	33.3 ± 26.8	40.3 ± 36.4	3.11	0.029

* Means: when the current expected count value was less than 5, the Fisher exact test was used to correct *p* value.

**Table 2:** Chest CT imaging manifestations of patients with NTM in four groups (*n*, %).

CT Manifestations	*M. intracellulare* Group (224 Cases)	*M*. *abscessus* Group (138 Cases)	*M*. *kansasii* Group (84 Cases)	*M*. *avium* Group (36 Cases)	*χ^2^* Value	*p* Value
Stripe shadow	151 (67.4)	91 (65.9)	46 (54.8)	28 (77.8)	7.07	0.070
Patchy shadow	154 (68.8)	114 (82.6)	70 (83.3)	31 (86.1)	14.40	0.002
Tree-in-bud sign	93 (41.5)	56 (40.6)	22 (26.2)	12 (33.3)	6.87	0.076
Nodular shadow	183 (81.7)	114 (82.6)	58 (69.0)	27 (75.0)	7.48	0.058
Pleural thickening or pleural effusion	138 (61.6)	93 (67.4)	32 (38.1)	20 (55.6)	19.94	<0.001
Mediastinal lymph node enlargement or calcification	37 (16.5)	29 (21.0)	6 (7.1)	4 (11.1)	8.25	0.041
Emphysema, bullae	86 (38.4)	36 (26.1)	29 (34.5)	12 (33.0)	5.80	0.122
Bronchiectasia	170 (75.9)	116 (84.1)	29 (34.5)	28 (77.8)	69.34	<0.001
Cavity	128 (57.1)	54 (39.1)	72 (85.7)	19 (56.6)	46.38	<0.001
Centrilobular tubercle	95 (42.4)	57 (41.3)	29 (34.5)	13 (36.1)	1.90	0.593
Number of involved lung fields (>3)	186 (83.0)	97 (70.3)	35 (41.7)	25 (69.4)	51.10	<0.001

## Data Availability

The datasets used and/or analyzed during the current study are available from the corresponding author on reasonable request.

## References

[1] Daley C.L., Iaccarino J.M., Lange C., Cambau E., Wallace R.J., Andrejak C., Böttger E.C., Brozek J., Griffith D.E., Guglielmetti L. (2020). Treatment of nontuberculous mycobacterial pulmonary disease: An official ATS/ERS/ESCMID/IDSA clinical practice guideline. Eur. Respir. J..

[2] Griffith D.E., Aksamit T., Brown-Elliott B.A., Catanzaro A., Daley C., Gordin F., Holland S.M., Horsburgh R., Huitt G., Iademarco M.F. (2007). An official ATS/IDSA statement: Diagnosis, treatment, and prevention of nontuberculous mycobacterial diseases. Am. J. Respir. Crit. Care Med..

[3] Haworth C.S., Banks J., Capstick T., Fisher A.J., Gorsuch T., Laurenson I.F., Leitch A., Loebinger M.R., Milburn H.J., Nightingale M. (2017). British Thoracic Society guidelines for the management of non-tuberculous mycobacterial pulmonary disease (NTM-PD). Thorax.

[4] Bryant J.M., Grogono D.M., Greaves D., Foweraker J., Roddick I., Inns T., Reacher M., Haworth C.S., Curran M.D., Harris S.R. (2013). Whole-genome sequencing to identify transmission of Mycobacterium abscessus between patients with cystic fibrosis: A retrospective cohort study. Lancet.

[5] Bryant J.M., Grogono D.M., Rodriguez-Rincon D., Everall I., Brown K.P., Moreno P., Verma D., Hill E., Drijkoningen J., Gilligan P. (2016). Emergence and spread of a human-transmissible multidrug-resistant nontuberculous mycobacterium. Science.

[6] DeFlorio-Barker S., Egorov A., Smith G.S., Murphy M.S., Stout J.E., Ghio A.J., Hudgens E.E., Messier K.P., Maillard J.M., Hilborn E.D. (2021). Environmental risk factors associated with pulmonary isolation of nontuberculous mycobacteria, a population-based study in the southeastern United States. Sci. Total Environ..

[7] Raju R.M., Raju S.M., Zhao Y., Rubin E.J. (2016). Leveraging Advances in Tuberculosis Diagnosis and Treatment to Address Nontuberculous Mycobacterial Disease. Emerg. Infect. Dis..

[8] Shahraki A.H., Heidarieh P., Bostanabad S.Z., Khosravi A.D., Hashemzadeh M., Khandan S., Biranvand M., Schraufnagel D.E., Mirsaeidi M. (2015). “Multidrug-resistant tuberculosis” may be nontuberculous mycobacteria. Eur. J. Intern. Med..

[9] Sarro Y.D., Kone B., Diarra B., Kumar A., Kodio O., Fofana D.B., Achenbach C.J., Beavogui A.H., Seydi M., Holl J.L. (2018). Simultaneous diagnosis of tuberculous and non-tuberculous mycobacterial diseases: Time for a better patient management. Clin. Microbiol. Infect. Dis..

[10] Kim H.O., Lee K., Choi H.K., Ha S., Lee S.M., Seo G.H. (2019). Incidence, comorbidities, and treatment patterns of nontuberculous mycobacterial infection in South Korea. Medicine.

[11] Xu D., Han C., Wang M.S., Wang J.L. (2018). Increasing prevalence of non-tuberculous mycobacterial infection from 2004–2009 to 2012–2017: A laboratory-based surveillance in China. J. Infect..

[12] Winthrop K.L., Marras T.K., Adjemian J., Zhang H., Wang P., Zhang Q. (2020). Incidence and Prevalence of Nontuberculous Mycobacterial Lung Disease in a Large U.S. Managed Care Health Plan, 2008–2015. Ann. Am. Thorac. Soc..

[13] Brode S.K., Daley C.L., Marras T.K. (2014). The epidemiologic relationship between tuberculosis and non-tuberculous mycobacterial disease: A systematic review. Int. J. Tuberc. Lung Dis. Off. J. Int. Union Against Tuberc. Lung Dis..

[14] Park S.C., Kang M.J., Han C.H., Lee S.M., Kim C.J., Lee J.M., Kang Y.A. (2019). Prevalence, incidence, and mortality of nontuberculous mycobacterial infection in Korea: A nationwide population-based study. BMC Pulm. Med..

[15] Adjemian J., Olivier K.N., Seitz A.E., Holland S.M., Prevots D.R. (2012). Prevalence of nontuberculous mycobacterial lung disease in U.S. Medicare beneficiaries. Am. J. Respir. Crit. Care Med..

[16] Tan Y., Su B., Shu W., Cai X., Kuang S., Kuang H., Liu J., Pang Y. (2018). Epidemiology of pulmonary disease due to nontuberculous mycobacteria in Southern China, 2013–2016. BMC Pulm. Med..

[17] Lee M.R., Chang L.Y., Ko J.C., Wang H.C., Huang Y.W. (2020). Nontuberculous mycobacterial lung disease epidemiology in Taiwan: A systematic review. J. Formos. Med. Assoc. Taiwan Yi Zhi.

[18] Morimoto K., Hasegawa N., Izumi K., Namkoong H., Uchimura K., Yoshiyama T., Hoshino Y., Kurashima A., Sokunaga J., Shibuya S. (2017). A Laboratory-based Analysis of Nontuberculous Mycobacterial Lung Disease in Japan from 2012 to 2013. Ann. Am. Thorac. Soc..

[19] Zweijpfenning S.M.H., Ingen J.V., Hoefsloot W. (2018). Geographic Distribution of Nontuberculous Mycobacteria Isolated from Clinical Specimens: A Systematic Review. Semin. Respir. Crit. Care Med..

[20] Sadamatsu H., Takahashi K., Tashiro H., Kusaba K., Haraguchi T., Kurihara Y., Komiya N., Nakashima C., Nakamura T., Kimura S. (2021). A Low Body Mass Index Is Associated with Unsuccessful Treatment in Patients with Mycobacterium avium Complex Pulmonary Disease. J. Clin. Med..

[21] Andréjak C., Almeida D.V., Tyagi S., Converse P.J., Ammerman N.C., Grosset J.H. (2015). Characterization of mouse models of Mycobacterium avium complex infection and evaluation of drug combinations. Antimicrob. Agents Chemother..

[22] Chu H., Li B., Zhao L., Huang D., Xu J., Zhang J., Gui T., Xu L., Luo L., Zhang Z. (2015). Tree-in-bud pattern of chest CT images for diagnosis of Mycobacterium abscesses. Int. J. Clin. Exp. Med..

[23] Park H.K., Koh W.J., Shim T.S., Kwon O.J. (2010). Clinical characteristics and treatment outcomes of Mycobacterium kansasii lung disease in Korea. Yonsei Med. J..

[24] Johnston J.C., Chiang L., Elwood K. (2017). Mycobacterium kansasii. Microbiol. Spectr..

[25] Matveychuk A., Fuks L., Priess R., Hahim I., Shitrit D. (2012). Clinical and radiological features of Mycobacterium kansasii and other NTM infections. Respir. Med..

[26] Zhao J., Jiang C., Wang Y., Wang H. (2009). Evaluate the application value of tuberculosisi antibody and enzyme-linked immuno-spot assay used for tuberculosis. Chin. J. Antituberc..

[27] Zhang S., Yang C., Fan L. (2018). Analysis of the diagnostic efficacy of serum tuberculosis antibody in active tuberculosis. Chin. J. Antituberc..

[28] Kwon Y.S., Koh W.J. (2016). Diagnosis and Treatment of Nontuberculous Mycobacterial Lung Disease. J. Korean Med. Sci..

[29] Zhou L., Xu D., Liu H., Wan K., Wang R., Yang Z. (2020). Trends in the Prevalence and Antibiotic Resistance of Non-tuberculous Mycobacteria in Mainland China, 2000–2019: Systematic Review and Meta-Analysis. Front. Public Health.

[30] Mirsaeidi M., Farshidpour M., Allen M.B., Ebrahimi G., Falkinham J.Q. (2014). Highlight on advances in nontuberculous mycobacterial disease in North America. BioMed Res. Int..

[31] Cheng L.P., Chen S.H., Lou H., Gui X.W., Shen X.N., Cao J., Sha W., Sun Q. (2022). Factors Associated with Treatment Outcome in Patients with Nontuberculous Mycobacterial Pulmonary Disease: A Large Population-Based Retrospective Cohort Study in Shanghai. Trop. Med. Infect. Dis..

[32] Weng Y.W., Huang C.K., Sy C.L., Wu K.S., Tsai H.c., Lee S.S. (2020). Treatment for Mycobacterium abscessus complex-lung disease. J. Formos. Med. Assoc..

